# Analysis of *In Vitro* Osteoblast Culture on Scaffolds for Future Bone Regeneration Purposes in Dentistry

**DOI:** 10.1155/2019/5420752

**Published:** 2019-02-06

**Authors:** Sandra J. Gutiérrez-Prieto, Sandra J. Perdomo-Lara, José M. Diaz-Peraza, Luis Gonzalo Sequeda-Castañeda

**Affiliations:** ^1^Dental Research Center, School of Dentistry, Pontificia Universidad Javeriana, Bogotá, Colombia; ^2^Basic Oral Research Unit, School of Dentistry, Universidad El Bosque, Bogotá, Colombia; ^3^Department of Physics, School of Sciences, Universidad Nacional de Colombia, Bogotá, Colombia; ^4^Department of Chemistry, School of Sciences, Pontificia Universidad Javeriana, Bogotá, Colombia

## Abstract

One of the main focuses of tissue engineering is to search for tridimensional scaffold materials, complying with nature's properties for tissue regeneration. Determining material biocompatibility is a fundamental step in considering its use. Therefore, the purpose of this study was to analyze osteoblast cell adhesion and viability on different materials to determine which was more compatible for future bone regeneration. Tridimensional structures were fabricated with hydroxyapatite, collagen, and porous silica. The bovine bone was used as material control. Biocompatibility was determined by seeding primary osteoblasts on each tridimensional structure. Cellular morphology was assessed by SEM and viability through confocal microscopy. Osteoblast colonization was observed on all evaluated materials' surface, revealing they did not elicit osteoblast cytotoxicity. Analyses of four different materials studied with diverse compositions and characteristics showed that adhesiveness was best seen for HA and viability for collagen. In general, the results of this investigation suggest these materials can be used in combination, as scaffolds intended for bone regeneration in dental and medical fields.

## 1. Introduction

Bone regeneration has increasingly turned into a very promising field, with use of resorbable biomaterials in combination with cells [1]. Tissue engineering works towards developing alternative treatments for autogenous or allogeneic bone and cartilage transplants [2–4]. Prospective tissue engineering applications aim at diminishing treatment complexity and decreasing healthcare system costs. Under optimal conditions, different cells can be cultured and seeded on natural or synthetic resorbable biomaterials used as scaffolds, which are then placed on the site where regeneration is needed. Furthermore, bioactive proteins delivered in matrix systems can signal cell differentiation and growth [5].

Scaffolds act as templates mimicking the functions of the extracellular matrix (ECM), where cells can interact and differentiate into their native phenotype. It is then necessary for the scaffold to meet the following conditions: the scaffold must be biocompatible, nontoxic, nonimmunogenic, easy to elaborate, and biodegradable. In addition, it must allow for proper cell survival and signaling. Moreover, cells must be capable of growth, cellular remodeling, and reorganization [6, 7]. Hence, ultimate scaffold properties mainly depend on the material's nature, its processing, and other material specifications with which it will interact.

One of the most important characteristics of the scaffold is a high interconnected porosity to enable vascularization for nutrient and gas diffusion, which permits waste disposal [8–10]. Additionally, some researchers point out the pore size should be 200 to 400 microns [7, 11, 12] and others of 500 microns [13]. Very small pores are obstructed by cells, preventing cell growth and bone matrix mineralization [14].

Thus, a proper porosity can improve mechanical retention between the scaffold and the host's environment, achieved as a result of ECM unspecified protein adsorption through a formed layer. This allows for a response from the cells in regard to the biomaterial they are in contact with, thus enabling new tissue formation [15]. Rustom et al. [16] and Babaie and Bhaduri [17] pointed out an optimal scaffold architecture is obtained through manufacture of a material with different pore sizes (micro and macro). The material must mimic the cancellous and cortical bone, for properly guided bone growth from within to cover bone defects.

Another aspect to consider is scaffold mechanical resistance to load and forces. The scaffold must provide properties as close as possible to the host's natural environment, to be degraded only when the new tissue is properly formed [12]. Currently, a combination of biocompatible materials has been proposed, in order to obtain better scaffold properties, which leads to adequate bone formation [18]. Various material alternatives, as well as various processing techniques, have been proposed for tissue engineering. It has been demonstrated that arrays of hydroxyapatite and polylactic acid-coglycolic increase bone formation [19]. Likewise, it has been suggested hydroxyapatite mixed with other materials may have therapeutic use for the treatment of tumors [20–22]. Other studies indicate that hydroxyapatite combined with polymers, such as chitosan and hyaluronic acid, has been used for drug transport and release [23].

Also, porous silicon seems promising for scaffold use in bone repair, since this material has been shown to promote osteoblast adherence and initiate maturation process [24, 25]. Furthermore, dissolution of bioactive glasses seems to elicit a genetic control effect on the osteoblast cycle, contributing to bone mineralization [26–29]. Additionally, silicon also seems to improve the mechanical properties and bioactivity of hydroxyapatite [24, 30]. Recent studies describe combination of organic and inorganic phases to create hybrid materials; such practices provide these materials with exceptional properties that allow them to have multiple functions and be used in various conditions [31].

In spite of these discoveries, the ideal scaffold for bone regeneration has not been found. Hence, the objective of this work was to analyze osteoblast attachment and viability on four different materials: bovine bone (control), hydroxyapatite (HA), porous silicon, and collagen to determine a possible scaffold fabrication candidate for bone regeneration.

## 2. Materials and Methods

### 2.1. Hydroxyapatite Synthesis

Hydroxyapatite synthesis was carried out by the wet technique. In a three-necked flask, 900 mL of 0.33 M calcium nitrate tetrahydrate (Ca(NO_3_)_2_·4H_2_O) was placed with 1,500 mL 0.12 M diammonium hydrogen phosphate ((NH_4_)_2_HPO_4_) at 1.0 mL/min. To adjust the pH to 12, 75 mL of ammonium hydroxide (NH_4_OH) was added. Subsequently, the solution was heated at 90°C, stirred for 1 hour, and incubated for 10 days at room temperature. The obtained precipitate was washed several times with distilled water to neutralize the pH, dried at 250°C for 1 h, and then calcinated at 1,000°C for 3 h.

The bovine bone was acquired as follows: samples of bovine femur were purchased from a local market. The bone was cleaned and disinfected to remove tissues, organic material, and microorganisms. Hot water was used for bone marrow extraction. Two percent NaOH was employed to remove fat and protein and 1% antiseptic solution (sodium hypochlorite) to prevent microorganism growth. Subsequently, the bone was rinsed with water to remove traces of cleaning and disinfecting solutions. It was then subjected to heat treatment at 1,000°C for 3 h to remove all organic materials [32].

### 2.2. X-Ray Diffraction (XRD)

XRD was performed on HA and bovine bone samples. X-ray diffractograms were obtained on a PANalytical X'Pert PRO MPD (Netherlands) device with the following parameters: measuring range between 2° and 90° (2*θ*), pitch size 0.02° (2*θ*), time per step 0.4 S, CuK*α*1 radiation *λ* = 1.5406 Å, and LynxEye detector. Crystalline phase qualitative identification present in the samples was carried out by comparison between reflections of the profile measured with diffraction profile reflections reported by the International Center for Diffraction Data (ICDD) (Powder Diffraction File) using search-match software [32].

### 2.3. Porous Silicon Synthesis

**Figure 1:**
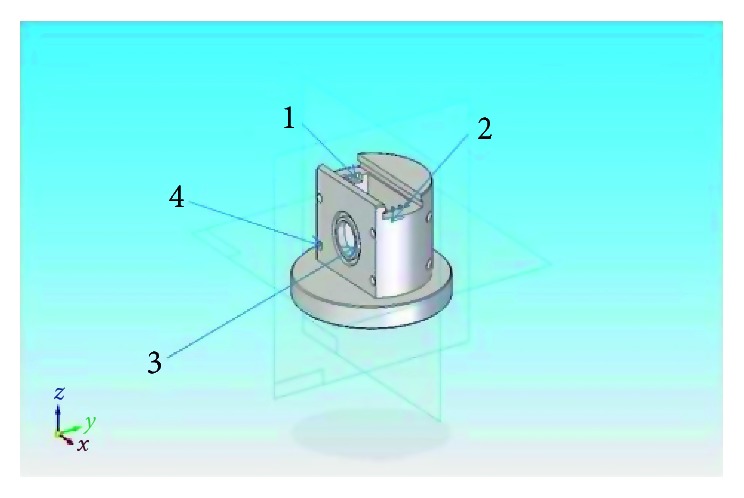
Cell design for porous silica elaboration (Professor Díaz-Peraza). Platinum electrode cell bracket (no. 1), p-type silicon acting as a second electrode (no. 2), and covering the circular orifice (no. 3) on a flat wall. The outer portion has a thin silicon layer of aluminum, previously vaporized to improve electrical contact between the silicon and an aluminum plate secured to the cavity by four screws (no. 4). Between the two electrodes, a DC current source was added and a milliamperimeter was used to supply and measure current.

Porous silicon was prepared by electrolysis using HF diluted with ethanol (HF : C_2_H_5_OH) (Merck KGaA) concentration [1 : 2] and a cell specially designed for this purpose (Figure 1).

### 2.4. Collagen Synthesis

Bovine tendon from the tail was selected for type I collagen extraction using 0.05 M acetic acid and continuous stirring for 72 h at 4°C. The obtained solution was subsequently filtered through a sterile gauze and centrifuged at 3,000 rpm for 2 h at 4°C. Finally, type I collagen solution was lyophilized, extracted, and stored at 4°C for subsequent 3D matrix construction.

### 2.5. Osteoblast Isolation and Cell Culture

Primary osteoblasts from the knee trabecular bone were obtained from the tissue obtained from joint replacement surgery, after informed signed consent by the patient. Surgery was performed at San Ignacio Hospital (Department of Orthopedics of Pontificia Javeriana University in Bogotá, Colombia) and approved by Research Ethics Committee at the Pontificia Universidad Javeriana act 002 of 2009.

Osteoblasts were isolated, following the method reported by Ducheyne and Qiu [33]. First, the trabecular bone was separated from the cortical bone. The trabecular bone was cut into pieces of approximately 1 to 2 mm. The bone fragments were treated with 2 mg/ml collagenase type II (300 U/mg) for two hours in a shaking water bath at 37°C. Explants were washed with sterile saline (PBS, pH 7.0) to remove hematopoietic cells and adherent cells from the bone marrow. Fragments were placed in 25 cm culture flasks, with the Dulbecco's Modified Eagle Medium: Nutrient Mixture F-12 (DMEM/F12; Gibco, Life technologies) supplemented with 10% fetal clone I (HyClone, Thermo Fisher Scientific), 100 U/ml penicillin, 100 *µ*g/ml streptomycin (Gibco, Life technologies), and 1.25 *µ*g/ml fungizone (Gibco, Life technologies) and incubated at 37°C and 5% CO_2_. The medium was changed twice a week until cells reached confluence. After seven days, cells migrating out of the tissue were visualized. The cells obtained were characterized by RT-PCR for osteocalcin (OCN), osteopontin (OPN), alkaline phosphatase (ALP), and bone sialoprotein (BSP) gene expression.

### 2.6. *In Vitro* Osteoblastic Evaluation Biocompatibility on the Different Materials

Primary human osteoblasts seeded into 12-well plates at a cell density of 50,000 cells/well on HA, silicon, bovine bone, and collagen matrices were evaluated for biocompatibility. To this end, manufactured porous matrices were sterilized with ethylene oxide and 1 × 10^6^ osteoblasts were seeded on each material. Cells were maintained in DMEM supplemented with 10% FBS and 200 *μ*g/ml penicillin/streptomycin for 7 days, followed by scanning electron microscopy (SEM) and fluorescent confocal scanning for biocompatibility evaluation.

### 2.7. Scanning Electron Microscopy (SEM)

After one week, culture matrices with cells were fixed with 3% buffered glutaraldehyde solution, followed by dehydration with increasing ethanol concentrations (50, 60, 70, 80, 90, and 100%), and dried at a critical point ending with gold coating. Morphology was evaluated using SEM on a JEOL Model JSM-6490 LV operated under high vacuum mode, equipped with sensors allowing for scattered electron imaging.

### 2.8. Fluorescent Confocal Scanning Evaluation

Cell viability was assessed on matrices (*n* = 4) by fluorescence microscopy using the live/dead kit (Invitrogen, Carlsbad, CA USA). This kit discriminates living cells, by the presence of ubiquitous esterase activity, which is determined by the enzymatic conversion of nonfluorescent calcein into intensely fluorescent calcein. The polyanionic dye calcein is retained inside living cells, producing an intense green fluorescence (excitation at 495 nm and emission at 515 nm). Furthermore, if plasma membrane integrity is lost, then ethidium homodimer-1 (EthD-1) enters the cells. An increase in bright red fluorescence is observed in dead cells (excitation at 495 nm with emission at 635 nm) caused by EthD-1 binding to nucleic acids. Staining was performed on the 3D matrix seeded without additional procedures and cut with a microtome.

## 3. Results

### 3.1. Elaboration of Scaffolds

**Figure 2:**
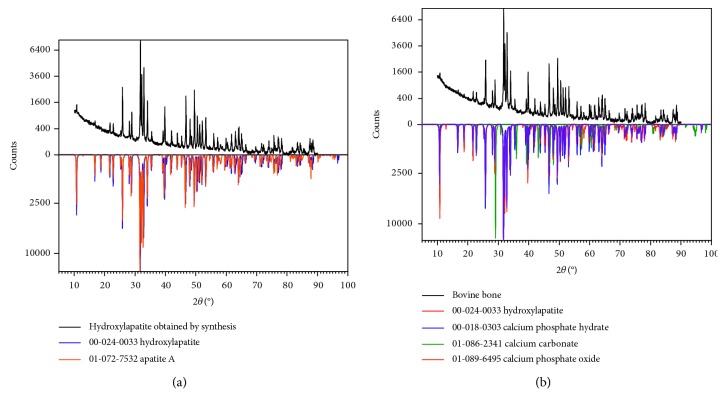
X-ray diffractograms of synthetic HAp and bovine bone. Qualitative identification of the crystalline phase established by comparison between reflections of the profile measured with diffraction profile reflections, reported by the International Center for Diffraction Data (ICDD) (powder diffraction file) using search-match software illustrates (a) hydroxyapatite components synthesized by the wet technique and (b) bovine bone analysis after treatment for cell seeding.

Hydroxyapatite sample X-ray diffractgrams are illustrated in Figure 2. Good crystallinity was obtained, evidenced by strong and narrow peaks. Qualitative analysis for each diffractgram was based on reflections corresponding to the compounds identified from PDF-2 database of the International Center for Diffraction Data (ICDD). For the bovine bone sample, hydroxyapatite (00-024-0033), phosphate hydrate (00-018-0303), calcium carbonate (01-086-2341), and calcium phosphate oxide (01-089-6495) were identified. The synthetic HA sample contained hydroxyapatite (00-024-0033) and apatite A (01-072-7532) (Figure 2(a)).

**Figure 3:**
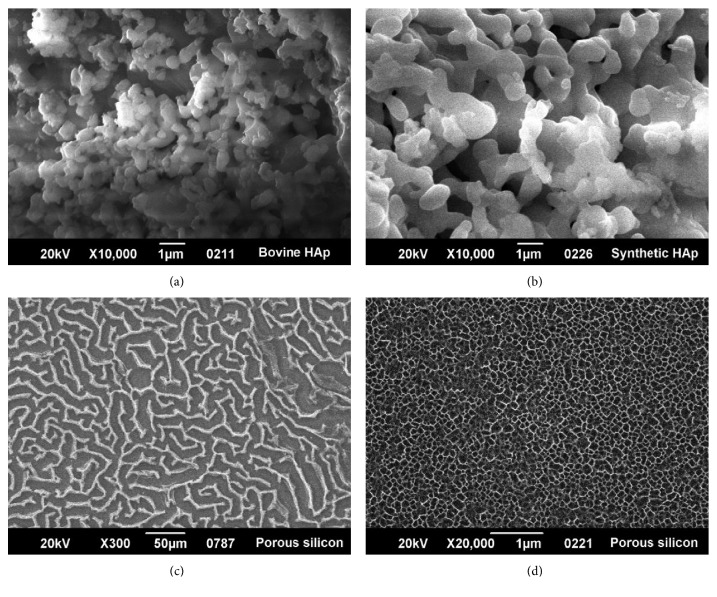
SEM micrographs of scaffolds used for osteoblast seeding. (a) Bovine bone (sintered at 1,000°C). Particles varied from smooth to rough, with asymmetric distribution. Shapes were round, irregular, short, or long. Other particles were polyhedral in configuration. Microporosity ranged in size range from 0.1 *μ*m to approximately 5 *μ*m. (b) Hydroxyapatite structure obtained by wet-precipitation method [32]. Globular grains were observed with different textures and sizes. Microscopic pores were polyhedral in shape and sizes varying between 1 and 3 *μ*m. (c) Porous silicon at 300X arranged in a labyrinth pattern. (d) Porous silicon at 20,000x illustrating very small pores of approximately 0.1 *μ*m.

Scaffolds prepared from four different materials were synthesized according to established methods, obtaining three-dimensional structures with different porosities and textures (Figures 3(a)–3(d)). Particles varied from smooth to rough, with asymmetric distribution, ranging from long, round, and soft irregular forms to short polyhedral particles. Microporosity ranged in size from 0.1 *μ*m to approximately 5 *μ*m. Globular grains were observed with different textures and sizes for hydroxyapatite obtained by wet precipitation (Figure 3(b)). Microscopic pores were polyhedral and varied in size between 1 and 3 *μ*m, similar to what was observed for bovine porous bone (Figure 3(a)). Porous silicon presented tiny pores arranged in a labyrinthine structure (Figures 3(c) and 3(d)). Collagen, showed fibrous, intertwined networks of long and parallel fibers (figure not shown).

### 3.2. *In Vitro* Osteoblastic Evaluation Biocompatibility on the Different Materials

#### 3.2.1. Scanning Electron Microscopy (SEM)

**Figure 4:**
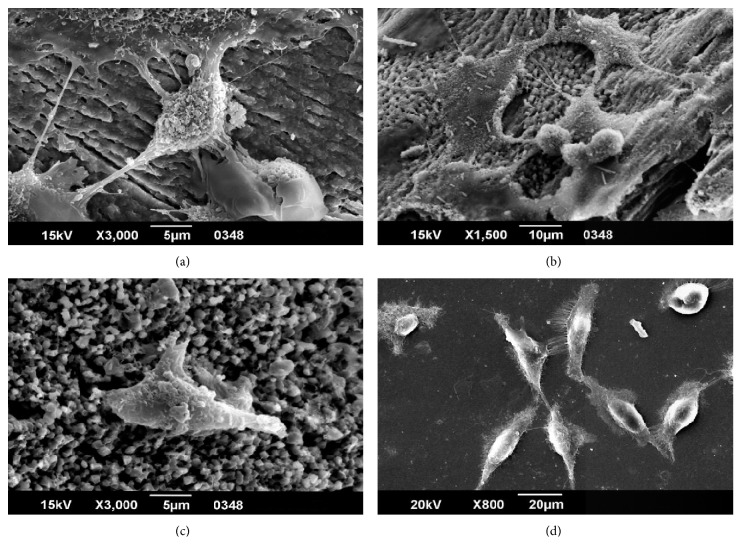
Osteoblasts' cell morphology on different scaffolds. SEM micrographs of osteoblasts seeded on different scaffolds and cultured for one week. (a) Osteoblasts seeded on bovine bone scaffold after seven days of culture at 3,000x. Cytoplasmic processes of osteoblast-substrate interactions and cell adhesion with filopodia and lamellipodia. (b) Osteoblasts seeded on bovine bone scaffold after seven days of culture at 1,500x. (c) Osteoblasts seeded on a HA scaffold after seven days in culture at 3,000x. (d) Osteoblasts seeded on a porous silicon scaffold after seven days of culture at 800x. Osteoblasts spreading on surface with filopodia in the substrate contact.

The results of SEM images showed osteoblast capability of cell attachment and interaction with other cells (Figures 4(a)–4(d)). After seven days of culture, osteoblasts seeded on bovine bone scaffolding had a healthy appearance with filopodia extending to other cells and the ECM (Figure 4(a)). In contrast to control (bovine bone), osteoblasts seeded on HA scaffold after seven days in culture did not reveal as much interaction with the scaffold as compared with control; namely, less extended filopodia were observed (Figure 4(c)). Even further, less interplay of cells with the matrix was observed for silica porous scaffold. Cells did establish interactions among them through extended filopodia (Figure 4(d)).

#### 3.2.2. Evaluation of Cell Viability

**Figure 5:**
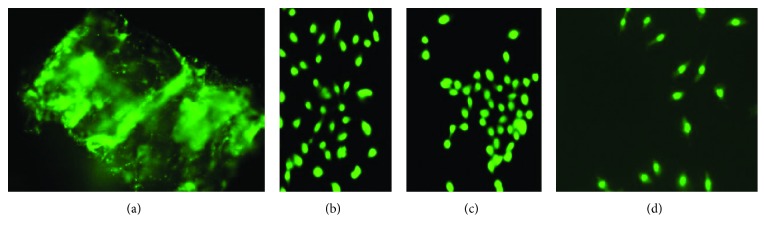
Primary osteoblast viability evaluation. (a) Osteoblasts seeded on collagen type I after seven days of culture. Fluorescence micrograph depicting viable cells as bright green dots immersed at multiple collagen sites. (b) Osteoblasts seeded on HA matrix after seven days in culture. (c) Osteoblasts seeded on bovine bone matrix after seven days in culture. (d) Fluorescence micrograph of osteoblasts on porous silicon after seven days in culture.

The results of fluorescence microscopy showed osteoblast viability in all four scaffolds after seven days of culture (Figures 5(a)–5(c)). Despite no dead cell signal, the number of cells diminished according to the material seeded. The highest number of cells was observed for the collagen scaffold (Figure 5(a)), bovine bone (Figure 5(c)), and HA (Figure 5(b)). The scaffold with the least number of cells observed was the silica scaffold (Figure 5(d)).

## 4. Discussion

Attractive alternatives to bone grafting have been achieved through recent advances in biomaterial development [34]. Many aspects should be taken into consideration for bone regeneration to occur by implementing tissue engineering techniques, among them cell interaction with scaffolds or matrices [35–37]. In addition, scaffolds must possess certain structural features to enable new tissue generation by cell growth and development on this specialized environment.

In this study, scaffolds were prepared with four different materials to evaluate *in vitro* osteoblast cell attachment and viability. The osteoblast primary culture was analyzed to determine if these materials could provide a suitable microenvironment. Criteria for material selection were based on (1) similarity to human bone characteristics, as was the control bovine bone; (2) structural component of nonorganic phase such as hydroxyapatite; (3) structural component of organic phase, i.e., collagen; and (4) last, a material with high mechanical strength characteristics such as porous silicon.

Hydroxyapatite has been one of the most used bioceramics in dental reconstruction and bone tissue regeneration due to its biocompatibility, osteoconductivity, and lack of cytotoxicity [9, 33]. However, HA has low mechanical strength and little degradation capacity [38, 39]. Nevertheless, its surface morphology resembles bone and because it is one of bone's nonorganic constituents, it is useful when mixed with other materials. This association is likely to contribute with increased bioactivity and biocompatibility. Additionally, other materials could improve hydroxyapatite shortcomings, such as mechanical strength.

Romanelli et al. developed a nanofibrous gel scaffold to which titanium nanoparticles and nanocrystals were added to simulate bone tissue, achieving osteoblast differentiation [40]. In this study, we used hydroxyapatite with morphological characteristics very similar to bovine hydroxyapatite, as shown in Figures 2 and 3 [32]. Viability and cell morphology results suggest this material could be used strategically to regulate cellular interactions and obtain good outcomes in clinical applications.

It is important to note that HA porosity can increase when combined with materials, such as gelatins, which supply HA ions improving cell viability but in turn decreasing its crystallinity and grain size [41]. Even though the obtained synthetic HA had no controlled porosity, in the present study, pores of a size varying between 1 and 3 *μ*m were obtained, which fall within the size range of bovine bone microporosities used as control (range from 0.1 *µ*m to approximately 5 *µ*m). It was identified that osteoblasts interacted with each other and adhered to the surface, extending their filopodia. This suggests HA could provide a suitable microenvironment for cell development. Furthermore, the technique can be improved allowing pore size control, which supports nutrient transport and toxin disposal [35, 42].

Another relevant aspect to consider is scaffold biodegradability. Several studies indicate use of HA mixtures with different materials to improve their slow degradation. Among these, Kasuya et al. used a mixture of phosphate cement gelatin/HA achieving improved mechanical properties, degradability, and bone formation [6]. In a study with rabbits, Kurashina et al. showed tricalcium phosphate (TCP) mixed with HA enabled faster degradation [43]. A recent publication reported on the HA composite with CaCO_3_ spheres injectable for bone repair use, with rapid degradation properties and new bone generation [44].

Recent studies evaluated the biodegradability and mechanical properties of a hydroxyapatite/chitosan/magnetite scaffold, showing that a higher hydroxyapatite content in the mixture and increased nucleation sites. This allowed increased mineral apposition by chitosan and magnetite, therefore increasing HA mechanical strength. On the other hand, biodegradability increased, with decreased HA content, due to decreased nucleation sites covering the surface, thus increasing the material's porosity. Collectively, a correlation between surface degradation and pore formation was observed [45].

In contrast, collagen has biodegradable properties and is a good alternative. Collagen type I has been considered as a promising scaffold material, since it is one of the most prevalent components of the ECM [46, 47]. This fibrous structural protein supports tension, pressure, and torsion [48]. The collagen obtained from bovine tail tendon was likely collagen type I, contributing with the necessary microenvironment for cell maintenance and tissue formation [27, 49]. However, given its fibrous nature, diffusion properties and cell migration possibility may be altered [35].

Won et al. [50] designed a scaffold, where collagen was introduced as a structural support. This novel proteinaceous hybrid contained osteocalcin-fibronectin fusion protein networked with fibrillar collagen. They demonstrated this hybrid material could be used as a potential scaffold for osteogenesis and bone regeneration [50]. The current literature reports show collagen scaffold fabrication mixed with hydroxyapatite (Col/HA), where a dual gradient of hydroxyapatite content was established that mimics the normal structure of the bone. Its structure was built in integrated layers, which allows formation of homogeneous and interconnected pores. These conditions allowed increase cell proliferation and osteogenic differentiation [51]. In the present study, it was observed that the collagen proved to be a matrix enabling osteoblast viability. In fact, it was the scaffold with the best results in this respect.

Porous silicon results showed osteoblast adhesion (Figure 4(d)). For this material, a roughened surface was formed by acid attack (Figure 1), which could have provided a favorable topography for cell adhesion. It has been shown that the surface texture is key to bioactivity properties. Based on our evidence, osteoblasts did adhere to the surface (Figure 4(d)). According to D'Elía et al., material bioactivity allows for cell adhesion, affecting the stability and lifetime of an implant or allowing for tissue regeneration [52]. In general, cell attachment is mediated by specific molecules, commonly integrins at specific binding sites. On surface attachment subjected to extracellular environment, nanodimensioned integrin receptors aggregate together and mobilize cytoplasmic proteins into a microfeatured complex, known as focal contact [53]. Upon adhesion to a substrate, the cell initially explores its environment and migrates using nanometer scale processes such as filopodia and lamellipodia [54].

Osteoblast adhesion on material surfaces is a long-term phenomenon, involving interactions among a host of biological molecules to induce signal transduction and cell response. The reactions of osteoblasts on material surfaces are exteriorized in a series of different time-related phenomena: protein adsorption at the material's surface, followed by a sequence of rapid short-term events constituting the attachment phase and subsequent adhesion phase, followed by proliferation, migration, and phenotypic differentiation (Figure 4) [55].

Osteoblast adhesion to substrates is mediated via adhesion molecules. The cell-matrix adhesions mechanically interlink internal actin filaments and matrix, leading to the formation of focal adhesions, focal plaque, or focal contact. Such focal contacts are tenacious adhesion structures, which remain attached to the substratum, even after forceful cell detachment (Figure 4) [56].

Collectively, all materials assayed provided different properties that need further optimization to achieve a substrate mimicking a natural environment. The best material providing a compatible surface for cell adhesion was bovine bone, followed by HA, porous silicon, and collagen. In contrast, after one week of culture, collagen sustained the most number of viable cells.

## 5. Conclusion

In this study, osteoblast cell morphology, adhesiveness, and viability were evaluated as a possible biocompatible indicator. We observed a favorable cellular response between the cells seeded onto the prepared scaffold. Depending on the substrate, different responses were observed. Adhesiveness was best seen for HA and viability for collagen. These materials have been widely reported as alternatives for 3D scaffold development [57–60]. This study represents an initial analysis that requires further evaluations to improve scaffold characteristics. Optimal conditions should enable cellular interaction, differentiation, biodegradation, and activation of signaling molecules promoting tissue regeneration.

Bone tissue engineering still holds many challenges. Scaffold material characteristics are key to reach the ultimate goal: cellular recognition, tissue specific commitment, repair, and regeneration. Therefore, it is of special interest to focus on using a combination of materials to provide mutual benefits. This interaction will tackle the weaknesses of one material by complementing with the strengths of the other material, leading to an ideal preparation for future scaffold establishment in tissue regeneration.

## Data Availability

The data used to support the findings of this study are included within the article.

## Supplementary Materials

Supplementary Materials The graphic abstract is included and is titled “SEM micrographs of structure and the CFM (confocal fluorescent Microscopy) images of cell viability, on different scaffolds after 7 days of osteoblastic cells culture.”
