# Supplementary figures and images for: Analysis of In Vitro Osteoblast Culture on Scaffolds for Future Bone Regeneration Purposes in Dentistry
Source: Adv Pharmacol Sci. 2019 Feb 6;2019:5420752. doi: 10.1155/2019/5420752 (PMC6381563; doi:10.1155/2019/5420752)

Graphical Abstract

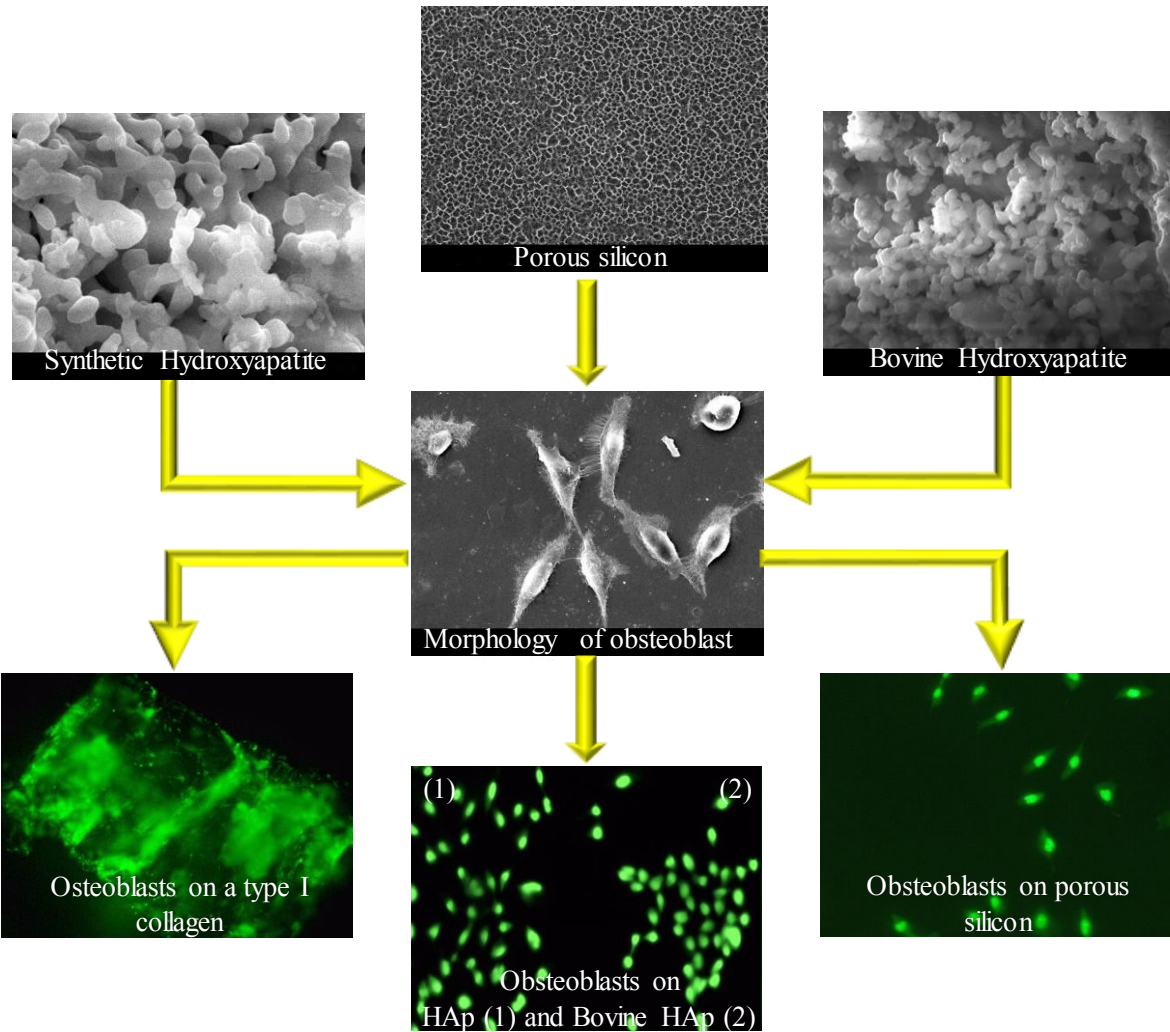

Supplement: Supplementary Materials — The graphic abstract is included and is titled “SEM micrographs of structure and the CFM (confocal fluorescent Microscopy) images of cell viability, on different scaffolds after 7 days of osteoblastic cells culture.” [file 5420752.f1.pdf]
